# The reciprocal interplay between TNFα and the circadian clock impacts on cell proliferation and migration in Hodgkin lymphoma cells

**DOI:** 10.1038/s41598-018-29847-z

**Published:** 2018-07-31

**Authors:** Mónica Abreu, Alireza Basti, Nikolai Genov, Gianluigi Mazzoccoli, Angela Relógio

**Affiliations:** 1 Charité - Universitätsmedizin Berlin, corporate member of Freie Universität Berlin, Humboldt - Universität zu Berlin, and Berlin Institute of Health, Institute for Theoretical Biology, Berlin, Germany; 2 Charité - Universitätsmedizin Berlin, corporate member of Freie Universität Berlin, Humboldt - Universität zu Berlin, and Berlin Institute of Health, Medical Department of Hematology, Oncology, and Tumor Immunology, Molecular Cancer Research Center, Berlin, Germany; 3 Department of Medical Sciences, Division of Internal Medicine and Chronobiology Unit, IRCCS “Casa Sollievo della Sofferenza”, San Giovanni Rotondo (FG), Italy

## Abstract

A bidirectional interaction between the circadian network and effector mechanisms of immunity brings on a proper working of both systems. In the present study, we used Hodgkin lymphoma (HL) as an experimental model for a type of cancer involving cells of the immune system. We identified this cancer type among haematological malignancies has having a strong differential expression of core-clock elements. Taking advantage of bioinformatics analyses and experimental procedures carried out in III- and IV-stage HL cells, and lymphoblastoid B cells, we explored this interplay and bear out diverse interacting partners of both systems. In particular, we assembled a wide-ranging network of clock-immune-related genes and pinpointed TNF as a crucial intermediary player. A robust circadian clock hallmarked III-stage lymphoma cells, differently from IV-stage HL cells, which do not harbour a properly functioning clockwork. TNF and circadian gene modulation impacted on clock genes expression and triggered phenotypic changes in lymphoma cells, suggesting a crucial involvement of core-clock elements and TNF in the physiopathological mechanisms hastening malignancy. Our results move forward our understanding of the putative role of the core-clock and TNF in the pathobiology of Hodgkin lymphoma, and highlight their influence in cellular proliferation and migration in lymphatic cancers.

## Introduction

Mammals, along with other species, have the ability to synchronize internal processes with changes in their environment. A circadian timing system regulates this synchrony and governs many aspects of cellular and behavioural physiology. These circadian rhythms allow organisms to anticipate daily light/dark cycles and are required to accommodate the 24 h pattern of rest and activity. The central pacemaker of the mammalian circadian system resides in the suprachiasmatic nucleus (SCN), and receives the light input from the external environment via the retinohypothalamic tract^1,2^. The central clock transmits signals to multiple peripheral biological clocks present in all cells. These oscillations (with a period of ~24 h) are tissue-specific and recent studies with mice revealed that about 50% of all genes show circadian expression^3^. At the molecular level, the core-clock network (CCN) consists of a set of 14 genes that form auto-regulatory positive and negative transcription-translation feedback loops^4^. These genes encode for members of PER (*Period* - *Per1*, *Per2*, *Per3*), CRY (*Cryptochrome - Cry1, Cry2*), BMAL (brain and muscle ARNT-like protein – *Bmal1*, *Bmal2*), CLOCK (NPAS2 in neuronal tissue) and the nuclear receptors gene and protein families ROR (*Rora*, *Rorb*, *Rorc*) and REV-ERB nuclear orphan receptor (*Rev-erbα*, *Rev-erbβ*)^5^. The heterodimerization of CLOCK/BMAL1 triggers its nuclear translocation and initiates the transcription of the feedback loops. In the first loop, the CLOCK/BMAL1 complex binds to E-box sequences in the promoter regions of *Per* and *Cry*. The PER/CRY heterodimer translocates to the nucleus and interacts with CLOCK/BMAL1 to inhibit transcription. The second loop regulates the expression of *Bmal1* by antagonistic effects of REV-ERB and ROR which compete for the ROR elements (RORE) in the *Bmal1* promoter. While RORs activate the expression of *Bmal*1, REV-ERBs repress it^6,7^.

Several biological processes are known to be under circadian regulation. These include the cell division cycle and DNA repair, RNA processing and transport, cellular metabolism, and the immune system functioning^4,8–14^. Notably, several key parameters of the immune system in the blood show circadian rhythms, which include the number of circulating hematopoietic and immunocompetent cells, as well as hormones, monoamines and cytokines. In fact, the communication between the circadian clock and the immune system appears to be bidirectional. The circadian clock influences elements of the immune system in its two branches: the innate branch (e.g. rhythmic cytokine expression and rhythmic monocyte and macrophage functions); and the adaptive branch (e.g. rhythmic T- and B-lymphocyte expression and count) of the immune system and is involved in immune disease models^15–18^. Interestingly, recent data show that the molecular clock controls metabolic pathways including immunometabolism and that the immune response (e.g. via TNF) could alter clock rhythmicity^19–21^.

Disruptions in the circadian clock are known to be associated with several types of diseases. In humans, several studies confirmed that circadian desynchronization in respect to the environment correlates with higher incidence of several types of cancer. These include non-Hodgkin lymphoma, breast, endometrial, prostate and colon cancers^8,22–26^. Furthermore, published data show that systemic inflammatory diseases are capable of altering the rhythmicity of several intrinsic signals. For example, effector cells in inflammatory reactions such as monocytes and neutrophils contain transforming growth factor (TGF-alpha) and release it upon activation^27^. TGF-alpha is rhythmically expressed in the mouse SCN, where it negatively impacts on locomotion control acting through the epidermal growth factor (EGF) receptor^28^. Moreover, the diurnal rhythms of leptin and cortisol secretion were lost in patients with acute sepsis^29^. Increasing evidence point to TNF (tumour necrosis factor) as a putative bridging element in the CCN/immune system complex. TNF is a multifunctional pro-inflammatory cytokine that belongs to the tumour necrosis factor superfamily and acts as a tumour repressor. Published data have shown the existence of circadian rhythms of TNF secretion in spleen cells stimulated with bacterial endotoxin and a loss of LPS-induced phase-delays of locomotor activity rhythms in *Tnfr1*-defective mice^30^. In particular, the expression of *Per1* was shown to be suppressed by TNFα in the human pancreatic cancer cell line MIA-PaCa2^31^. These findings illustrate the significant regulatory role of the CCN on the immune response and support the further development of new therapeutic approaches, entailing chronotherapy and other time-dependent intervention strategies. Despite the increasing relevance of the biological clock in cancer onset and progression, the role of key immune elements, such as TNF, in mediating clock dysregulation in lymphatic cancers remains elusive.

Here, we used Hodgkin lymphoma (HL) cells as a lymphatic cancer cell model, to explore the effects of clock dysregulation in an immune-related context, though the chosen experimental system cannot be generalized to infer circadian clock functionality in HL or in other haematological neoplastic diseases. Considering that HL is a type of cancer involving cells of the immune system (lymphocytes), as a first step we generated a comprehensive circadian clock/immune system network of genes that pointed to TNF as a major networking partner. To further investigate the interplay between lymphoid malignancies and the circadian clock, in our disease model, we knocked-down (KD) several core-clock genes, including *Bmal1*, *Per2* and *Rev-erbα* and analysed the effects in terms of changes in gene expression and cell phenotype (cell cycle phase, proliferation, apoptosis and migration).

Additionally, in our lymphatic cancer model, we investigated the role of TNF as a potential interacting partner between mutated pathways and the circadian clock. We stimulated WT and KD cells with TNF, as well as generated *Tnf* KD cell lines and analysed the effects on the clock phenotype and the cell cycle. Our findings from a combined experimental-bioinformatics approach suggest that in our model of lymphatic cancer the circadian clock impacts on the malignant phenotype and TNF acts as a major interacting partner for the circadian clock affecting key cellular functions.

## Results

### Immuno-clock network and clock signature in Hodgkin lymphoma

**Figure 1:**
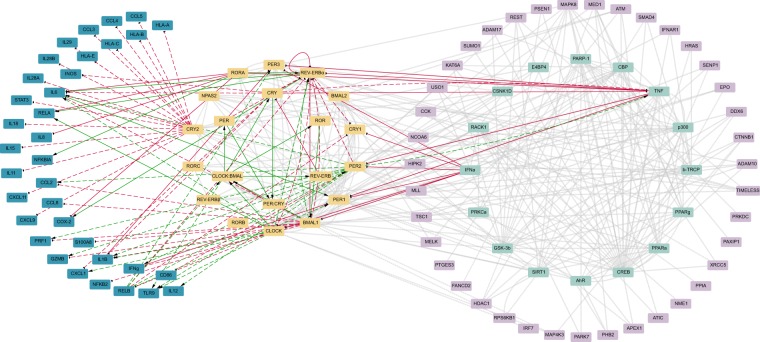
The network of clock-immune related genes (NCIRG) connects elements of the circadian clock network and the immune system. An enrichment analysis on immune-related GO terms and KEEG pathways of the NCRG, and manual curation based on published data of immune-related genes with interactions with the CCN added a set (35) of immune-related genes (dark blue) to the network. Orange, light green and light purple elements represent the core-clock network (CCN, 20 elements), the extended core-clock network (ECCN) and the network of clock-related genes (NCRG), respectively. Grey lines represent the connection between genes previously published in the NCRG; green and red lines refer to positive and negative (respectively) interactions between newly added immune-related genes and the CCN, based on published data. Black arrows indicate the direction of each interaction. Dashed lines mark the interactions found in modified *in vitro*/*in vivo* models. It is important to notice that in some of the studies used for the construction of the network double KD or KO animals/cells were used. In such cases, it was not possible to specify the exact gene responsible for the reported effect on the immune genes, CRY, PER, ROR and REV-ERB were used to refer to the gene family. The direction of the effect is represented by an arrow for activation and a dead end arrow for the inhibition pointing to the affected gene.

The circadian clock regulates several behavioural and physiological processes in mammals among which the immune response^32^. We used a previously generated network of circadian-regulated genes (NCRG)^4^ as the starting point for the construction of a comprehensive network of elements (genes, proteins, and protein complexes) which couple the core-clock to the immune system. The NCRG was derived from an extended core-clock network (ECCN) which represents a regulatory network containing elements that have direct interactions with the core-clock. Based on high-throughput gene co-expression data analysis and text-mining tools we further extended the ECCN network to build the NCRG, further developed in this work. A subsequent enrichment analysis for immune-related terms resulted in a selection of 16 genes from the ECCN and additional 39 genes from the NCRG. The selected genes were enriched in GO terms and/or KEGG immune-related pathways. We carried out a manual curation of published data on chrono-immunology, to complement the bioinformatics approach described above, which resulted in an additional list of 35 genes involved in immune function and response, and shown to be regulated by (or to regulate) the circadian clock. The newly generated regulatory network represents an up-to-date comprehensive bidirectional communication between the circadian clock and the immune system (Fig. 1, and Table S1).

The existence of a bidirectional communication between both systems, raises the interesting question regarding the putative role of this interplay in cancer. We aimed to choose an experimental cancer model of the immune system with differential expression of circadian clock elements. For this, we used high-throughput data from the Cancer Cell Line Encyclopaedia (CCLE)^33^ and analysed the expression of core-clock elements.

We selected expression data from 172 haematopoietic and lymphoid tissue cancer cell lines and compared the normalized expression data in a pair-wise manner, among the different cancer groups.

Subsequently, we carried out an analysis for enriched elements present in the pathway “Circadian rhythms related genes“ in the WikiPathway data base. This list was mostly enriched in comparisons between Hodgkin lymphoma (HL), a lymphoproliferative disease characterized by the presence of Hodgkin and Reed-Sternberg cells, and the other haematological cancers with 37 ± 4 (mean, SEM) differentially expressed genes within the defined pathway (Fig. S2A–D). This points to a severe dysregulation of circadian clock elements in HL, as compared to other tumours of the hematopoietic and immune system, which made this particular cancer type an interesting model system for our subsequent analysis.

### The Hodgkin lymphoma cell line HD-MY-Z shows robust oscillations in gene expression

We choose the HL cell lines HD-MY-Z (HL stage III b) and L-1236 (HL stage IV) to model disease progression and a lymphoblastoid B-cell line (LCL-HO) (established by EBV transformation, considering the recognized pathogenetic role for EBV infection in HL). There are relevant clinical differences between the different stages in Hodgkin lymphoma (HL). In stage III HL, the disease involves lymph node areas on both sides of the diaphragm or lymph nodes above the diaphragm and in the spleen. In stage IV HL, the disease has spread widely into at least one organ outside of the lymph system. In the presence of additional symptoms e.g. loss of more than 10% of body weight, fever and/or drenching night sweats, the letter B is added to the stage^34^.

**Figure 2:**
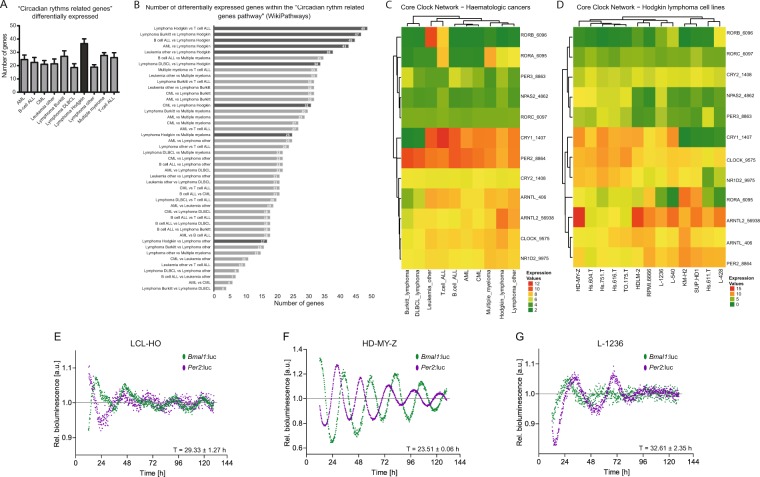
Cancer cell line encyclopedia (CCLE) analysis revealed a circadian signature in Hodgkin lymphoma cell lines. (**A**) Represented is the number of genes, belonging to the “Circadian rhythms related genes” (WikiPathway), differentially expressed in the several haematopoietic and lymphoid tissue cancer cell lines present in the database. (**B**) Individual comparisons between the groups of cell lines regarding the number of differentially expressed genes of the pathway referred in A. (**C**,**D**) Heatmaps for relative expression levels of CCN genes in haematopoietic cancers and Hodgkin lymphoma cell lines, respectively. (**E**,**G**) LCL-HO, HD-MY-Z and L-1236 have differential circadian phenotypes. Shown are bioluminescence readouts for the promoter activity of *Bmal1* (green) and *Per2* (purple) over the course of 144 hours in LCL-HO (T = 29.33 ± 1.27 h; phase *Bmal1* = 18.67 ± 0.3 h) (**E**) and the HL cell lines HD-MY-Z (T = 23.51 ± 0.06 h; phase *Bmal1* = 11.64 ± 0.26 h) (**F**) and L-1236 (T = 32.61 ± 2.35 h; phase *Bmal1* = 20.15 ± 0.3 h) (**G**). Periods and phases were calculated with ChronoStar software (n = 3, mean ± SEM).

The three cell types exhibit different oscillatory patterns of core-clock gene expression as depicted by non-invasive long-term bioluminescence recording and expression of the core-clock genes, *Bmal1* and *Per2* (Fig. 2E–G). LCL-HO (T = 29.33 ± 1.27 h*;* n* = *3*;* mean and SEM) and L-1236 (T = 32.61 ± 2.35 h*;* n* = *3*;* mean and SEM) showed weak oscillations for both genes with prolonged periods (representative results in Fig. 2E,G), thus suggesting a disruption in the circadian clock machinery in the mentioned cell lines. Furthermore, the antiphasic oscillation pattern between *Bmal1* and *Per2* is partially lost for these cell lines. In contrast, the HL cell line HD-MY-Z showed a strong circadian clock with robust (T = 23.51 ± 0.055 h; n* = *3*;* mean and SEM; representative results in Fig. 2F) and antiphasic oscillations of *Bmal1* and *Per2*. We subsequently focused on HD-MY-Z cells as a HL model and the EBV growth-transformed lymphoblastoid LCL-HO cell line as a model system of EBV latency and virus-driven B cell proliferation and tumorigenesis. In these cell lines, we further analysed the expression levels for *Bmal1* and *Per2* via RT-PCR in the course of 24 hours, and these were in agreement with the bioluminescence data (Fig. S1A,B).

**Figure 3:**
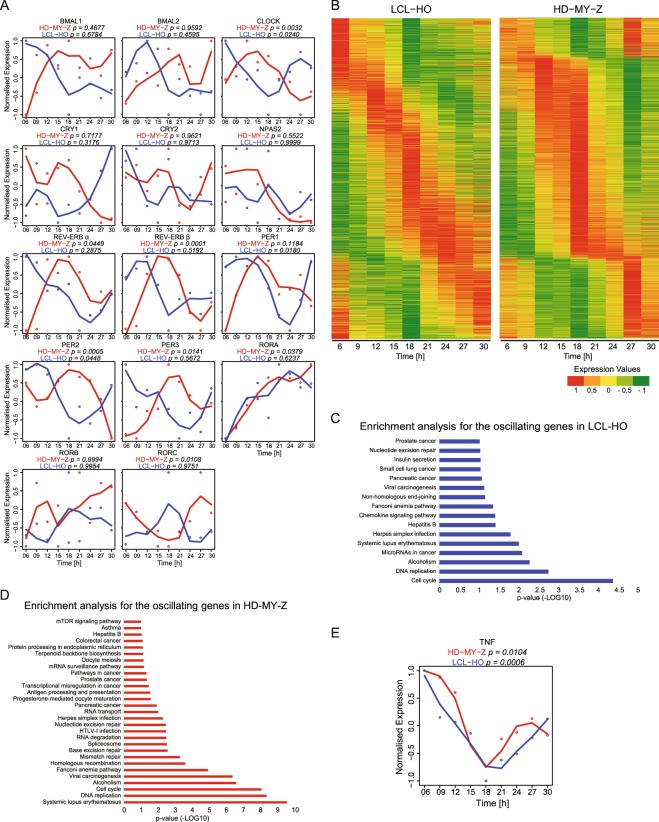
The genome-wide landscape of oscillating genes in LCL-HO and HD-MY-Z revealed cell-specific circadian regulation in HL. (**A**) Depicted are expression values for core-clock genes for LCL-HO (blue) and HD-MY-Z (red) retrieved from the time course array data. (**B**) Oscillating genes present in LCL-HO and the HL cell line HD-MY-Z, respectively, phase ordered and normalized to values between −1 and 1. (**C**,**D**) Enrichment analysis (KEGG pathways) for the previously described genes in the different cell lines. (**E**) TNF relative gene expression retrieved from the array data in LCL-HO (p = 0.0006) and HD-MY-Z (p = 0.0104), respectively. (**A**,**E**) Based on the data from the time course HTA2.0 microarray experiments we performed an additional normalization in the range 1, −1. The normalized data was used to fit a curve with Local Polynomial Regression Fitting. The genes were analysed with the MetaCycle R package for a period set to 21 to 27 hours. The combined Metacycle p-values are displayed for each gene.

To investigate the overall presence of circadian oscillating genes in the LCL-HO and the HL cell line HD-MY-Z, we carried out a genome wide time-course array analysis. Out of all genes, 55% (13825) were differentially expressed between both cell lines including a set of genes enriched for the TNF signalling pathway (*P* = 5.18E-10) (Fig. S2A–C). Among those, 7402 genes (29%) were downregulated in the HL cell line and 6495 (26%) were upregulated, as compared to the lymphoblastoid B-cells. Our enrichment analysis indicated the predominance of cancer-related pathways within the set of upregulated genes, and immune-related pathways for the set of downregulated genes. Interestingly, the circadian rhythm pathway is enriched in the HL cell line HD-MY-Z, in the set of upregulated genes (*P* = 0.0117) (Fig. S2D,E). The strong upregulation of the circadian rhythm pathway prompt us to analyse the expression levels of the CCN genes for both cell lines in more detail (Fig. 3A). In particular, the expression levels of *Cry1* and *Npas2* were strikingly different between the two cell lines (Fig. S1C). This finding was also confirmed by RT-qPCR, which showed more than a 2-fold increase in the expression of *Cry1* and *Npas2* in the HL cell line HD-MY-Z as compared to the lymphoblastoid B-cells LCL-HO (Fig. S1D). Both *Cry1* and *Npas2* (a paralog of *CLOCK*) belong to core-clock components and regulate the negative and positive feedback loops, respectively. They are involved in DNA damage response and cell cycle, and are important for maintaining the correct circadian rhythm of the cells. Next, we searched for commonly/differentially oscillating genes in both cell lines. As expected, the HL cell line HD-MY-Z, which showed a robust circadian clock, had a higher number of oscillating genes than the lymphoblastoid B- cell line LCL-HO (7462 and 1925 oscillating genes respectively, Fig. 3B). Notably, there were 746 genes oscillating in both cell lines. Both subsets of oscillating genes showed similar enriched pathways, with a predominance of cancer-related terms (Fig. 3C,D). *Tnf* oscillated with significant values in both cell lines (LCL-HO, *P* = 0.003; HD-MY-Z, *P* = 0.01) as depicted in Fig. 3E and it was also differentially expressed in both cells. TNF signalling was one of the most enriched pathways among the downregulated genes in HD-MY-Z (Fig. S2D). This corroborated a role for TNF as a modulator of the circadian clock in our cellular model of a cancer type involving cells of the immune system.

### TNF stimulation alters CCN gene expression and cell cycle phase

**Figure 4:**
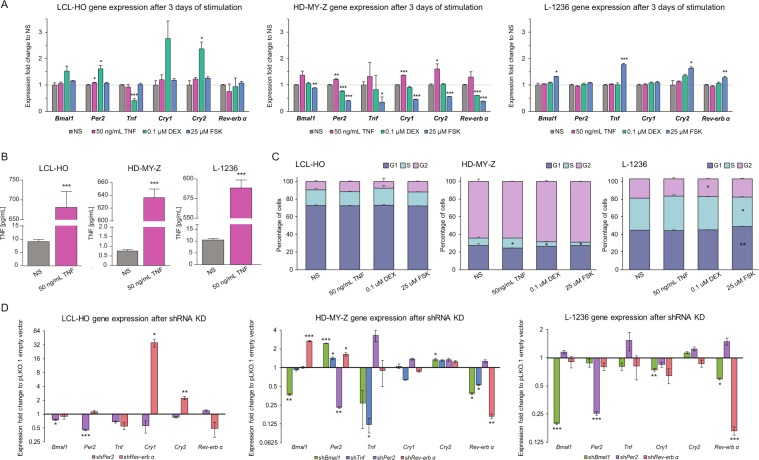
TNF affects the cell cycle and the expression of core-clock genes in HL. Gene expression, supernatant concentration of TNF and cell cycle analysis for the three cell lines (WT) after three days of stimulation with 50 ng/mL of recombinant human TNF, 0.1 uM dexamethasone and 25 uM of Forskolin. (**A**) Gene expression qPCR data (n = 3, mean ± SEM). (**B**) Supernatant concentration of TNF (n = 3, mean ± SEM). (**C**) Cell cycle analysis for the three cell lines (LCL-HO, the HL cell line HD-MY-Z and the HL cell line L-1236) (n = 3, mean ± SEM). (**D**) Gene expression data for *Bmal1*, *Per2*, *Tnf, Cry1*, *Cry2* and *Rev-erbα* of LCL-HO, HD-MY-Z and L-1236 cell lines after shRNA KD (n = 3, mean ± SEM). The corresponding shRNA KD for the specific cell is represented under each plot. ^*^p < 0.05, ^**^p < 0.01, ^***^p < 0.001.

TNF is a potent pro-inflammatory cytokine responsible for the regulation of immune cells. This cytokine was also reported as a regulator of genes of the CCN^35,36^. To gain a deeper understanding of the role of TNF as an elicitor of the core-clock, we stimulated the HL cell lines HD-MY-Z and L-1236 (HL, stage-IV), which showed weaker oscillations compared to the HD-MY-Z cell line (HL stage-III, Fig. 2F,G), with TNF for three days and measured gene expression levels of the core-clock genes *Bmal1, Per2, Cry1, Cry2, Rev-erbα*, as well as *Tnf* (Fig. 4A). The stimulations were carried out at a specific time point, but the cells remained in the presence of the stimulating agent during the three days (Materials and Methods) so we no longer had a single time point effect, but a constant effect, as compared to the non-stimulated condition. Our results indicated a general increase in the expression of all core-clock genes in the HL cell line HD-MY-Z upon TNF stimulation, with *Per2*, *Cry1* and *Cry2* showing significant changes (*P* < 0.01, *P* < 0.001 and *P* < 0.05 respectively). In contrast, in the HL cell line L-1236 after a three-day stimulation with TNF there were no changes observed in the mRNA expression levels of the mentioned genes. Additionally, we carried out stimulations with dexamethasone and forskolin, to introduce clock perturbations. Dexamethasone is a known potent anti-inflammatory agent used to treat inflammatory and autoimmune diseases^37^. It is also used in many circadian studies to synchronize cells in culture^38^. Like dexamethasone, forskolin is also used to synchronize the cellular clock^38^ and in addition it has a known inhibitory effect on *Tnf*^39^. Here we stimulated the two HL cell lines, and the lymphoblastoid B-cell LCL-HO with dexamethasone and forskolin for three consecutive days, and evaluated the resulting phenotype in the clock machinery, as well as in *Tnf* (Figs 4A, and S3A–E, S4A–E). Furthermore, we additionally examined the impact of TNF stimulation on TNF and IL-6 secretion in the three cell lines (Figs 4B, S5). Interleukin-6 (IL-6) is secreted by HL cells, throws in the immunoreactive phenotype and plays a significant pathobiological role in this lymphatic cancer^40^. As expected, the levels of extracellular TNF increased in all cell lines upon stimulation. Interestingly, the two cell lines showing weaker oscillations (lymphoblastoid B-cell LCL-HO and the HL cell line L-1236, Fig. S5), had higher levels of both TNF and IL-6 in the supernatant as compared to the HL cell line HD-MY-Z. Next, we analysed the possible impact of TNF stimulation on the cell cycle in the three cell lines (Figs 4C, and S3D, S4E) and observed a significant change for the HL cell line HD-MY-Z, which resulted in an increased ratio of cells in the S phase, suggesting a proliferative role of TNF in this cell line.

### TNF downregulation influences the expression of core-clock elements

We performed shRNA KD in CCN elements and in *Tnf* to evaluate the output in terms of gene expression, cell proliferation, extracellular concentration of TNF, cell cycle, apoptosis and migration. Upon sh*Bmal1* KD, the HL cell line HD-MY-Z showed a significant increase in the expression of *Per2* and *Cry2* and a decrease in *Rev-erbα*, as well as a decrease in *Tnf* expression (Fig. 4D). Additionally, sh*Tnf* caused alterations in gene expression of *Per2* and *Rev-erbα*, increasing and decreasing their expression, respectively, in the HL cell line HD-MY-Z, while the KD of *Rev-erbα* led to an increase in the expression of *Bmal1* and *Per2* (Figs 4D and S6). *Per2* and *Rev-erbα* silencing exhibit different and specific effects on the expression of core-clock genes in the three cell lines used in this work. In LCL-HO, only *Rev-erbα* downregulation caused significant alterations in *Cry1* and *Cry2* increasing their expression, which is in agreement with the known negative regulation of *Rev-erbα* on *Cry* expression. In the HL cell lines, we observed an increase in *Per2* and *Bmal1* expression after *Rev-erbα* downregulation. Additionally, the KD of *Per2* led to an increase in *Tnf* expression, in the HL cell line HD-MY-Z. In the HL L-1236 cell line however, we could not detect significant changes in the expression of core-clock genes and *Tnf* both in sh*Per2* as in the sh*Rev-erbα* cell lines. For the HL cell line L-1236, we observed similar results for *Rev-erbα* gene expression in the sh*Bmal1* cells, but the expression levels of *Per2* and *Cry2* remained unchanged. In the same cell line, the expression of *Cry1* was significantly decreased (Fig. 4D). The KD of *Bmal1* led to cell death in the LCL-HO cell line (Fig. S3E). Taken together, these results suggest a major role of TNF in the regulation of several key core-clock genes.

### Effects of TNF stimulation on key cellular characteristics of the KD cell lines

**Figure 5:**
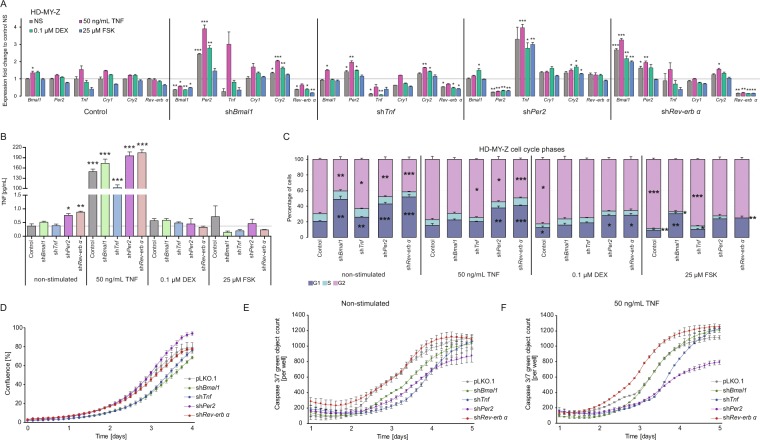
Dysregulation of the core-clock and of T*nf* led to differential cell cycle phenotypes in the HL cell line HD-MY-Z. (**A**) Gene expression qPCR data (n = 3, mean ± SEM). (**B**) TNF concentration in the supernatant (n = 3, mean ± SEM). (**C**) Cell cycle measurements after KD of *Bmal1*, *Tnf*, *Per2* and *Rev-erbα* followed by stimulation for 3 days with 50 ng/mL of recombinant human TNF, 0.1 µM dexamethasone and 25 µM of forskolin (n = 3, mean ± SEM). (**D**) Proliferation analyses of HD-MY-Z cell line after shRNA KDs. (**E**,**F**) Apoptosis analysis of HD-MY-Z cell line after KDs in both non-stimulated and TNF (50 ng/ml) stimulated conditions, respectively (n = 3, mean ± SEM). Statistics for the comparison to non-stimulated (NS) control ^*^p < 0.05, ^**^p < 0.01, ^***^p < 0.001.

Next, we aimed to explore the stimulatory effects of TNF in the expression of core-clock genes (*Bmal1, Per2* and *Rev-erbα)*, as well as in the expression of *Tnf* in the KD cell lines. The KD cells were stimulated for three days with 50 ng/mL of human recombinant TNF, 0.1 µM of dexamethasone or 25 µM of forskolin. The stimulation of the HL cell line HD-MY-Z with human recombinant TNF triggered an increase in gene expression of the evaluated genes in all the considered experimental conditions (Fig. 5A). When compared to the non-stimulated control (non-stimulated, empty vector), the main differences were found in *Per2* expression for sh*Bmal1* cells, *Tnf* expression in sh*Per2* cells and *Bmal1* expression in sh*Rev-erbα* cells. Additionally, the downregulation of *Tnf* caused an increase in *Per2* expression and a decrease in *Rev-erbα* expression. We observed an increase in the expression of *Bmal1* and *Per2* in sh*Rev-erbα* cells. We further observed an increase in the extracellular concentration of TNF for sh*Per2* and sh*Rev-erbα* cells, suggesting an inhibitory effect of *Per2* and *Rev-erbα* on TNF secretion (Fig. 5B). Furthermore, given the known correlations between IL-6 and TNF expression and also the clock^41,42^, we aimed to explore whether changes in the expression of TNF in different KD cell lines and after TNF stimulation impacts IL-6 expression (by measuring the IL-6 concentration in the supernatant). An increase of IL-6 concentration was also observed in LCL-HO sh*Rev-erbα* cell line (Fig. S3).

We further analysed the impact on the cell cycle caused by perturbations in the core-clock, as well as in *Tnf* and observed an increase in the percentage of cells in G1 phase and decrease in G2 phase of the cell cycle for the different knockdowns in the HL cell line HD-MY-Z (Fig. 5C). We did not observe a significant effect on the cell cycle in sh*Tnf* and sh*Rev-erbα* cells upon stimulation with TNF, as compared to the observations in the non-stimulated scenario, however, sh*Bmal1* cells acquired a profile closer to the non-stimulated control and the sh*Per2* cells showed an increase in the G2 cell cycle phase. Despite the similar output in terms of *Tnf* inhibition, dexamethasone and forskolin did not have similar effects on gene expression of core-clock genes and cell cycle, in the HL cell line HD-MY-Z. Forskolin decreased the *Per2* and *Cry2* expression in the sh*Bmal1* cells (Fig. 5A). In HD-MY-Z-sh*Tnf* cells, we observed a decrease in *Per2* gene expression and an increase in *Tnf*, after forskolin treatment. Both dexamethasone and forskolin increased *Per2* expression in HD-MY-Z-sh*Per2-*cells, however, forskolin also showed an effect on *Bmal1*, increasing its expression. In sh*Rev-erbα* cells treated with forskolin there was a decrease in *Per2* expression. Dexamethasone treatment led to an increase in G2 and decrease in G1 phases in the HL cell line HD-MY-Z as compared to non-stimulated cells, and increased G1 phase in sh*Per2* and sh*Rev-erbα* conditions (Fig. 5C). Similar to dexamethasone, forskolin also increased G2 and decreased G1 cell cycle phases as compared to non-stimulated cells, however these effects were more prominent. Cell cycle profiles of sh*Tnf* and sh*Rev-erbα* cells showed alterations after forskolin stimulation, which reverted the effects caused by the shRNA towards a profile similar to the non-stimulated cells (Fig. 5C). In sh*Per2* cells, forskolin caused an increase in G2 with a consequent decrease in G1, as compared to the non-treated HD-MY-Z-sh*Per2*. Finally, we examined the impact of the different knockdowns on cell proliferation, for the HL cell line HD-MY-Z (Fig. 5D). Compared to the pLKO.1 (empty vector), sh*Per2* cells showed increased proliferation, whereas *shBmal1* and sh*Tnf* dramatically reduced proliferation (Fig. 5D).

### Dysregulation of the circadian clock impacts HL cell migration and apoptosis

We further analysed the influence of circadian clock dysregulation in the HL cell line HD-MY-Z regarding apoptosis and migration. Our data showed a remarkable decrease in apoptosis in sh*Per2* and sh*Tnf* HD-MY-Z-cells and an increase of apoptosis in the sh*Rev-erbα* cells (Fig. 5E). To investigate the role of TNF in apoptosis, we stimulated all KD cell lines with TNF and measured their apoptotic rate. Upon TNF stimulation, we observed a general increase in the apoptosis rate of sh*Rev-erbα-*, sh*TNF- and* sh*Bmal1-*HD-MY-Z cells, corroborating a tumour suppressing role for TNF (Fig. 5F). Interestingly, sh*Per2* cells exhibited a greater decrease in apoptosis compared to the non-stimulating scenario (Fig. 5E,F). This points to an inhibitory effect of TNF on *Per2* expression resulting in reduced apoptosis.

**Figure 6:**
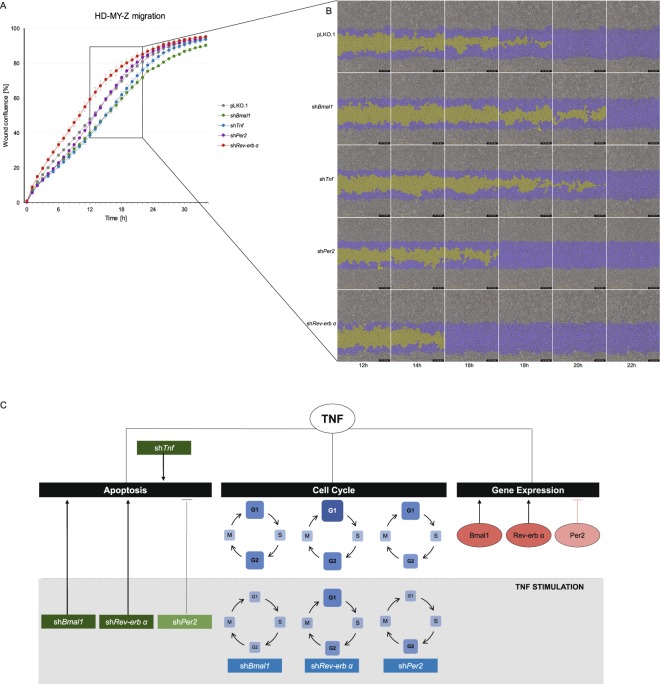
*Tnf* and core-clock genes affected cell migration in the HL cell line HD-MY-Z. (**A**) Migration properties of pLKO.1 empty vector and KD HD-MY-Z cell lines (*shBmal1, shTNF, shPer2* and *shRev-erbα*), measurements were obtained using a scratch wound assay for the IncuCyte S3 Live Cell System Analysis. Quantification was performed by measuring the relative wound confluence over the course of 34 hours. (n = 3, mean ± SEM). (**B**) Partial representation of the scratch wound assay for all HD-MY-Z KD derived cell lines in the course of 12–22 hours. Images were obtained with the IncuCyte S3 Software. Yellow mask indicates the wound border locations. Blue mask indicates the initial scratch wound area. (**C**) A model for the bi-directional role of TNF and the core-clock in regulating the molecular and cellular characteristics in HL. TNF regulates gene expression, cell cycle and apoptosis by interacting with the core-clock elements *Bmal1*, *Per2* and *Rev-erbα* (orange circles). The grey area represents stimulatory effects of TNF on KD cell lines. TNF stimulation differentially affects the cell cycle phases of the KD cell lines, as illustrated by the different sizes and colour of each blue square (compared to pLKO.1). Cellular apoptosis is influenced by TNF stimulation in shRNA cell lines leading to its activation (sh*Bmal*, sh*Rev-Erb)* or inhibition (sh*Per2*).

We additionaly explored the impact of the different perturbations in the migratory properties of the HL cell line. Cell migration was determined by measuring the wound confluence over 34 hours (Fig. 6A,B). Among all KD cell lines, sh*Rev-erbα* showed the highest migration activity when compared to the empty vector control cells, suggesting that suppression of *Rev-erbα* expression had a positive effect on the migration properties of the HL cell line HD-MY-Z. In contrast, sh*Tnf* and sh*Bmal1* cell lines showed decreased migration when compared to the empty vector control cells. These results indicate that CCN genes, as well as TNF are involved in key cellular properties of HL cells, and their influence in cell proliferation and migration may play a role in HL progression.

## Discussion

The circadian clock machinery generates rhythmic patterns in the expression of genes and proteins that allow physiological and cellular functions to be fine-tuned and coordinated in time. These are crucial for the timely segregation of incompatible cellular processes and for energy conservation purposes. This preserved rhythmicity characterizes also the function and components of the immune system, and ensures the conservation of body homeostasis and defence mechanism. Several studies point to a bidirectional crosstalk between the circadian pathway and the immune system, and characterize the effects of inflammation and cytokines on circadian rhythms^43,44^.

In this study, we further investigated the extent of such a bidirectional communication in a lymphatic cancer and validated different interacting partners of both systems by generating a comprehensive network of clock-immune related genes (NCIRG) which highlighted a major potential bridging element, TNF. To examine this interplay, we first carried out an enrichment analyses for circadian rhythm related genes in different cancers and found Hodgkin lymphoma to have a significant differential expression for genes of the circadian rhythm pathway.

From the analysis of high-throughput time-course data for a stage-III HL cell line (HD-MY-Z) and by comparing these results with the lymphoblastoid-B cell line, LCL-HO, which exhibits weak oscillations in the expression of core-clock genes, we determined the number of oscillating genes in both cell lines and found a strong link between the oscillating genes and cancer-related pathways, in particular for the HL cell line HD-MY-Z. Since we identified more robust circadian oscillations and a larger number of oscillating genes in the HL cell line HD-MY-Z with respect to the LCL-HO cell line, we suggest that a properly functioning circadian clock machinery might be necessary for HD-MY-Z cell survival and malignant phenotype. *Tnf* is a well-known immune system gene. Its expression is controlled by the core-clock network via the D-box elements found in its promoter region^45^. TNF and its receptors (TNFR1 and TNFR2) fulfil several functions, namely the crucial control of lymphocyte expansion and preservation of their structural integrity, and function as well as immunocompetent cells in the development of immune responses. TNF seems to act as a bridging element between the core-clock and the immune system, as also shown by our data. TNF modulates the immune system function through the down-regulation of T-cell receptor (TCR) signalling, calcium flux changes, p21, p23 and ZAP70 activation, and reduced NF-κB nuclear activation^41^. Conversely, the biological clock directly controls the production of important cytokines: in particular CRY1 and CRY2 suppress the expression of the genes encoding TNFα and IL-6, and REV-ERBα restrains the induction of IL-6. In turn, these cytokines impact the functioning of the biological clock^42^. IL-6 levels are augmented in patients with Hodgkin lymphoma and are correlated with a poor prognosis. Also in our study we measured an increase of IL-6 in the HL stage-IV cell line L-1236, as compared to the stage-III cell line HD-MY-Z. Furthermore, IL-6 and other cytokines are produced in HL by Reed-Sternberg cells or infiltrating reactive cells, comprising lymphocytes, plasma cells, eosinophils and histiocytes, and might contribute to the impairment of immune response and to leukocyte trafficking, preserving a supporting environment for RS cells survival. IL-6 acts in an autocrine or paracrine way supporting a tumour environment that allows for Reed-Sternberg cells survival and proliferation by hindering apoptosis and host anti-tumour defence, which is likely to trigger weight loss, night sweats, fever, and other paraneoplastic reported symptoms^46^. The appropriate modulation of IL-6 dependent pathways may consent to expand the targeted approaches in HL therapy^47^.

In our experiments, we inspected the oscillating pattern of *Tnf* expression in the HL cell line HD-MY-Z and the lymphoblastoid-B cells LCL-HO and investigated the role of TNF stimulation at the molecular and cellular levels in WT and knock-down variants of HD-MY-Z. Upon TNF stimulation, we observe a general increase in the expression levels of core-clock elements, among them *Bmal1*, *Per2* and *Rev-Erbα*. This finding is consistent with previous studies in rheumatoid synovial cells^36^ and in fibroblasts at early time points^48^ which point to elevated expression of the mentioned genes upon TNF stimulation. Following gene knock-down, we measured gene-specific effects of TNF on the core-clock elements. In particular, *Per2* expression appears to be negatively controlled by TNF. While the expression of *Per2* was significantly increased in sh*Tnf* cells, *Tnf* expression was increased dramatically in sh*Per2* cells. This inhibition could be due to E-box-mediated interfering effects of TNF on *Per2* transcription as previously reported^49^. In patients with metastatic colorectal cancer statistically significant correlations were found between pre-treatment levels of cytokines, circadian patterns in wrist activity and serum cortisol and tumour-related symptoms. In particular, patients with reduced 24-hour rest/activity patterns quantified by actigraphy showed significantly higher serum levels of TGF-α, TNF-α and IL-6^17^. Similarly, in our study we observe higher levels of TNF-α for the two cell lines which have the less robust clock (LCL-HO and the HL cell line L-1236, Fig. S5) further strengthening the previously described correlation between the clock phenotype and alterations in immune elements, in a cancer context.

Our results further indicate a role for TNF as an enhancer of proliferation leading to an increased amount of cells in the S-phase in HD-MY-Z cells (Fig. 6C) which can be achieved via the activation of the NF-kB pathway. Interestingly, we did not find significant effects of TNF stimulation in the cell cycle for LCL-HO and L-1236 cell lines indicating its specific role in cell lines which are hallmarked by proper oscillations of clock genes. As such, the loss of a functional clock in the L-1236 seems to affect the feedback from the TNF to the core-clock clock (upon perturbation of TNF the expression levels of the clock genes is rather constant), and to result in a larger S-phase, as observed for these cells. Interestingly, in non-Hodgkin lymphoma patients circadian rhythms in S-G2-M cell cycle phases were shown in malignant lymph nodes of stage IIIB (p = 0.046 for 24-h cosine fit), but not stage IV (p = 0.560 for 24-h cosine fit) disease^16^. Even though this is a different model (non-Hodgkin lymphoma) as compared to the one used in our work (Hodgkin lymphoma), we observed as well a loss of robustness in circadian rhythms for the stage IV cell lines. This could indeed impact the cell cycle given the strong interconnection between the cell cycle and the circadian clock oscillators, as shown by our own work and others^14,50^.

Altogether, our results obtained from KD cell lines, point to a role of TNF in regulating the cell cycle for HD-MY-Z KD cells, and its ability to restore altered cell cycle phases in KD cells. Our results from the proliferation analysis data identified *Per2* as a potential proliferation inhibitor, while *Bmal1* and *Tnf* acted as enhancers of cell proliferation in the HL cell line HD-MY-Z. This could result from the negative regulatory role of PER2 in the clock machinery and the positive effects of BMAL1 and TNF in enhancing the transcription of clock-controlled genes involved in proliferation. Importantly, *Bmal1* and *Per2* may impact cellular physiology in different ways. Either influencing the clock machinery and altering circadian rhythms, and/or by influencing the expression of genes that are direct targets of CLOCK/BMAL1 and/or of PER2. This could be a result of other potential, non-circadian, functions of *Bmal1* and *Per2*. To differentiate between these two hypotheses and to better characterize the specific output results of *Bmal1* and *Per2* knock-down phenotypes further studies are necessary.

Cell apoptosis was dramatically influenced and decreased in sh*Per2* cells upon TNF stimulation (Fig. 6C), which can be correlated with the inhibitory effects of TNF on *Per2*. Since TNF represses *Per2* expression, this could lead to a decreased expression of *Bmal1* resulting in reduced CLOCK/BMAL1 levels and eventually derepression of c-Myc followed by reduced apoptosis, as previously reported^51^. The migratory properties of the HL HD-MY-Z KD cell lines reveals a role for *Rev-Erbα* as a motility suppressor. These findings corroborate the results of experiments performed in an acute myeloid leukaemia mouse model showing that leukaemia stem cells are dependent on a functioning biological clock. Derangement of the molecular clockwork induces weakened proliferation, superior myeloid differentiation, and leukaemia stem cells exhaustion, with resulting overall anti-leukemic effects^52^. It would be interesting in future work to validate our results in an *in vivo* model (mouse, humans). However, this goes beyond the scope of this study that we consider exploratory in this first phase.

The interplay between the circadian and the immune systems highlighted by our bioinformatics analyses and corroborated by our experimental procedures performed in lymphoma cells pinpointed a crucial role played by this bidirectional interaction in determining cancer cell phenotype. According to our results, a deregulated biological clock impacts on proliferation, migration and cell death in HL cell lines. Differently from what has been observed for solid cancers, a well-running molecular clockwork ticks in medium stage lymphoma cells and supports malignancy, whereas its perturbation hinders malignant phenotype maintenance and it seems to be altered in more advanced lymphoma stages. Hodgkin lymphoma, as well as other lymphoma types, is not representative of the whole immune system, considering the monoclonality hallmarking cancer beyond intratumour heterogeneity in comparison to the multiclonality of the majority of immune components, and the genomic and phenotypic alterations of a cancer respect to the normal tissue. As such, additional model systems such as T-cell lymphoma, leukaemia and multiple myeloma should be considered in future work, to further extend the findings here reported to cancers of the immune system. Key players in the aforementioned interplay could be represented by small molecules, in particular cytokines such as TNF and IL-6, whose secretion is circadian regulated, and which have been shown capable to impact the functioning of the biological clock. In our study we focused on TNF, a major inflammatory cytokine originally documented as capable to provoke, in experimental cancers, haemorrhagic necrosis, but subsequently found to paradoxical act as a tumour-promoter. Our results corroborate its role as a key intermediary of the circadian - immune network, impacting the expression of core-clock genes and inducing phenotypic changes in lymphoma cells. The findings of our experimental approach may also contribute to advance our knowledge of the physiopathological mechanisms bringing on malignancy in lymphoma cells with the aim to identify key molecules and valuable compounds for potential targeted therapeutic strategies. Even though we did not explore the usage of therapeutics associated to the circadian system^53^ in this work, there are potential implications of our findings in the clinic which justify future work. Cell-autonomous molecular clockworks may play a significant role in lymphoid malignancies treatment impacting lymphatic cancer cells phenotype and in particular cell growth and proliferation, by driving oscillations of cell-cycle genes important for the G1-to-S transition. The cell division cycle is under circadian control and restoring clock function in lymphatic cancer cells harbouring a dysfunctional molecular clockwork could slow down their growth. In contrast, clock-interfering treatments such as inhibition of the cancer clock by shRNA-mediated knock-down, could significantly decrease proliferation and tumour growth in lymphatic cancer cells hallmarked by rhythmic clock gene expression. Characterization of the subjective patterns of oscillatory gene expression could orientate tailored therapy of lymphoma patients in the setting of personalized medicine. Furthermore, the investigation of the circadian control over the cell cycle through small molecules impacting the biological clock could lead to the modulation of lymphatic cancer cells proliferation and support anticancer effects of chemotherapy and surgical tumour resection.

## Methods

### Network of clock-immune related genes

The network construction involved two different approaches. In the first approach, an enrichment analysis of the previously published network - NCRG^4^ was carried out using DAVID Bioinformatics Resources 6.8^54^. Genes containing immune-related associations were selected for the present network. Interactions from the previous network were maintained and are represented in grey. Text mining was performed with the Agilent Literature Search plugin for Cytoscape using the Pubmed database. The second approach involved manual curation of published experimental data. Positive and negative interactions are represented in green and red, respectively. Network visualization was generated using Cytoscape version 3.4^55^. A list of new interactions can be found in Table S1.

### Cancer Cell Line Encyclopaedia (CCLE) analysis

Cancer Cell Line Encyclopaedia (CCLE) contains expression data from 947 cancer cell lines performed using Affymetrix GeneChip Human Genome U133 Plus 2.0 Array. A total of 174 cell lines were included in the CCLE tumour type group 2 (haematopoietic) and grouped into 10 different groups: acute myeloid leukaemia (AML, n = 33), B cell acute lymphoblastic leukaemia (B-cell ALL, n = 12), chronic myeloid leukaemia (CML, n = 14), T-cell acute lymphoblastic leukaemia (T-cell ALL, n = 14), others_leukaemia (n = 5), Burkitt’s lymphoma (n = 10), diffuse large B cell lymphoma (DLBCL, n = 16), Hodgkin lymphoma (HL, n = 13), others_lymphoma (n = 28) and multiple myeloma (n = 27). Robust Multi-array Average (RMA) normalisation was performed by the Expression Console (EC (Affymetrix)) and Gene level differential expressed analysis were performed by Transcriptome Analysis Console v3.0 (TAC (Affymetrix)). One-Way Between-Subject ANOVA (unpaired) algorithm was used to detect differential expressed genes (filter criteria: Fold Change (linear) <−2 or >2 and ANOVA p-value (condition pair) <0.05). Heatmaps were generated using ComplexHeatmap software package^56^ for statistical programing environment R. The number of differentially expressed genes in the “Circadian rhythm related genes” (WikiPathways^57^) was manually counted.

### Cell culture

HD-MY-Z (DSMZ – #ACC 346): established in 1991 from the pleural effusion (rich in Hodgkin/Reed-Sternberg cells) from a 29-year-old patient with nodular sclerosing Hodgkin lymphoma (stage IIIb) refractory to therapy. Semi-adherent spheroid to fusiform cells growing in clumps in mono- and multilayers. Doubling time: ca. 35 hours. L-1236 (DSMZ - #ACC 530): established from the peripheral blood of a 34-year-old man with Hodgkin lymphoma (mixed cellularity, stage IV, refractory, terminal, third relapse) in 1994. Semi-adherent spheroid to fusiform cells growing in clumps in mono- and multilayers. Doubling time: ca. 48 hours. LCL-HO (DSMZ – #ACC 185): B lymphoblastoid cell line, established by EBV transformation (Epstein-Barr virus). Lymphoblastoid-like cells growing in suspension singly or in clumps. Doubling time: ca. 30 hours.

HD-MY-Z, LCL-HO and L-1236 (Hodgkin lymphoma, stage IV, refractory, terminal, third relapse) cell lines were maintained in RPMI 1640 (Gibco) supplemented with 1% penicillin-streptomycin (Gibco) and 10% foetal bovine serum (Gibco). Stable-transduced cells were selected and maintained in medium containing 100 μg/mL hygromicin B (Gibco) for the *Bmal1*:luc hygromicin (BLH), 10 μg/mL of blasticidin S HCl (Gibco) for the *Per2*:luc blasticidin (PLB) and 10 μg/mL of puromycin for the shRNA KD of the clock genes and *Tnf*. For live-cell bioluminescence recording (evaluation/analysis/monitoring), cells were maintained in phenol red-free RPMI 1640 (Gibco) containing 10% foetal bovine serum, 1% penicillin-streptomycin and 0.1 mM D-Luciferin (PJK). For cell stimulation, cells were maintained for 3 days in medium (RPMI 1640 (Gibco) supplemented with 1% penicillin-streptomycin (Gibco) and 10% foetal bovine serum (Gibco)) with 50 ng/mL of human recombinant TNF (BioLegend), or 0.1 µM of Dexamethasone (Sigma-Aldrich) or 25 µM of Forskolin (Sigma-Aldrich) as previously described^58–62^. Cell morphology and concentration were analysed in LUNA™ Automated Cell Counter (Logos Biosystems). All cells were incubated at 37 °C with 5% CO_2_ atmosphere. All three cell lines express glucocorticoid, TNF and cathecolamine receptors as previously reported^63,64^ and as also fund in our array data.

### Lentivirus production

Lentiviral elements containing a BMAL1-promoter-driven luciferase (BLH), a PER2-promoter-driver luciferase (PLB), an empty vector (TRC Lentiviral pLKO.1 Empty Vector Control Dharmacon Inc., Lafayette, CO) or a shRNA KD (TRC Lentiviral Human ARNTL shRNA - Clone ID: TRCN0000019097; TRC Lentiviral Human TNF shRNA - Clone ID: TRCN0000003758; TRC Lentiviral Human PER2 shRNA - Clone ID: TRCN0000018541;TRC Lentiviral Human NR1D1 shRNA - Clone ID: TRCN0000022176 (Dharmacon Inc., Lafayette, CO)) were used in the present work. HEK293T (human, kidney, ATCC Number: CRL-11268) cells were seeded in 175 cm^2^ culture flasks and co-transfected with 12.5 µg packaging plasmid psPAX, 7.5 µg envelope plasmid pMD2G and 17.5 µg BLH,PLB or shRNA KD expression plasmid using the CalPhos mammalian transfection kit (Clontech) according to the manufacturer’s instruction. To harvest the lentiviral particles, the supernatant was centrifuged at 4100 × g for 15 min to remove cell debris and passed through a 45 µm filter. Lentivirus were stored at −80 °C.

### Transduction with lentiviral vectors

1 × 10^6^ cells were seeded in a 6-well plate with 1 mL of RPMI1640, 10% foetal bovine serum, 1% penicillin-streptomycin and 4 µg/mL Polybrene (Sigma). 1 mL of supernatant of the corresponding lentivirus were added to each well and plates were centrifuged for 90 min at 34 °C at 1000 g. Supernatant was discarded after 4 hours incubation at 37 °C with 5% CO_2_ and 2 mL of fresh medium was added. Two days after, the medium was replaced by selection medium (supplemented with hygromicin S, basticidin S HCl or puromycin (according to the lentivirus construct used)) and incubated at 37 °C with 5% CO_2_ atmosphere. Cells were maintained in selection medium for at least two weeks. For the shRNA KD, the KD efficiency was determined by gene expression analysis.

### Synchronization and measurement of circadian rhythms

For bioluminescence measurement, 0.5–1 × 10^6^ cells were washed once with 1× PBS, plated in 35 mm dishes and synchronized by adding fresh phenol-red-free RPMI 1640 medium supplemented with 10% foetal bovine serum, 1% penicillin-streptomycin and 0.1 mM D-Luciferin. *Bmal1*-promoter-(BLH)-reporter or *Per2*-promoter-(PLB)-reporter activities were measured, using the LumiCycle instrument (Actimetrics). After bioluminescence recording for 5–7 days, phase, period and amplitude were analysed with the ChronoStar analysis software V3.0 using 24 h running average de-trended method and fitting cosines curves were calculated as output^65^.

### RNA isolation and cDNA synthesis

Cells were seeded in 35 mm dishes with a density of 5 × 10^5^ cells/dish and synchronized by fresh medium addition. Cells were collected every three hours, in triplicates and the pellets were stored at −80 °C. Total RNA was isolated using the RNeasy extraction kit (Qiagen), including DNase digestion, according to the manufacturer’s instructions. Cells were lysed with 350 µl RLT buffer and the lysate was homogenized. Total RNA concentration was determined using NanoDrop® ND-1000 UV-Vis Spectrophotometer and stored at −80 °C. 1 µg of total RNA was reverse-transcribed to cDNA with M-MLV reverse transcriptase (Invitrogen), random hexamers (Eurofins MWG Operon) and dNTPs Mix (Thermo Fisher).

### Quantitative real-time PCR

For gene expression quantification, we used SYBR-Green (Bio-Rad Laboratories, Hercules, CA) based real-time PCR in 96-well plates and the human commercial primers from QuantiTect primer assays (Qiagen) for *Gapdh* (housekeeping gene), *Bmal1*, *Per2, Tnf, Cry1, Cry2* and *Rev-erbα*. The reaction was performed in a real-time PCR Detection System (Bio-Rad). Each sample was measured in duplicates and the C_T_ values were determined by using the regression method. The expression levels were normalized to those of *Gapdh* (ΔCT) and calibrated to the mean expression value of each gene (time-course analysis) or in relation to the respective control. (ΔΔC_T_). The relative quantification was performed using the 2^−ΔΔCT^ method.

### Microarray analysis

Microarray analysis was performed in R version 3.4.2 using the oligo package. Multi-Array Average (RMA) pre-processing procedure was used to normalise expression levels of genes^66^. HTA 2.0 were annotated by merging data with probeset annotations provided online (www.affymetrix.com). Heatmaps were generated using ComplexHeatmap software package^56^ for statistical programming environment R. The R package arrayQualityMetric^67^ was used for quality control and statistical testing of the arrays.

The microarray dataset has been deposited in the ArrayExpress database at EMBL-EBI (www.ebi.ac.uk/arrayexpress) under the accession number E-MTAB-6400 and will be released upon publication. Differentially expressed genes were determined with the limma package^68^. Normalization of the arrays was done for both cell lines together using RMA, the additional normalization of array data was done with the BBmisc R package in the range −1, 1. The MetaCycle R package was used to determine the oscillating genes with the three built-in methods ARS, JTK and LS. The period was set to 21–27 h. Only genes with a Metacycle combined p-value lower than 0.05 were used for further analysis.

### Enrichment analysis

The analysis in enriched pathways was performed using the KEGG data base. Enrichment analysis were performed in an open access online platform, DAVID Bioinformatics Resources 6.8^54^ using the default settings.

### ELISA

To detect the supernatant concentrations of TNF and IL-6, a “sandwich” enzyme-linked immunosorbent assay (ELISA) was performed using the Human TNF-α ELISA MAX™ Deluxe and Human IL-6 ELISA MAX™ Deluxe (Biolegend, San Diego, CA), respectively, according with the manufactorer’s instructions. Plates’ absorbance was read at 450 nm in VICTOR light 1420 (Perkin Elmer, Waltham, MA).

### Cell cycle measurements

Cells used for cell cycle studies were washed with 1X PBS and fixed with ice-cold 80% ethanol. After fixation, cells were washed, resuspended in 150 µL of 1X PBS and stained for 30 min with 150 µL of a solution containing 0.05 mg/mL of PI (Sigma-Aldrich), 0.3% triton x-100 (Sigma-Aldrich) and 10 mg/mL of RNase (AppliChem GmbH, Darmstadt, Germany) in 1X PBS. Samples were read in FACSCabilur (Becton Dickinson, Franklin Lakes, NJ). The cells of interest were gated based on forward scatter/side scatter (FSC versus SSC) values. The cell cycle analysis was handled by fitting a univariate cell cycle model to the gated population using the Dean-Jett Fox^69^ option, implemented in FlowJo v10.2 (FlowJo, LLC, Ashland, OR).

### Proliferation, apoptosis, and migration measurements

#### Proliferation assays

For the proliferation assay, 2500 cells/well were seeded in a 96-well plate (Eppendorf). Cell were allowed to adhere and placed in the IncuCyte® S3 Live Cell System Analysis (Essen BioScience). Four pictures were recorded every hour for the 3 biological replicates and 4 technical replicates. Analysis were performed using the IncuCyte S3 Software (Essen BioScience).

#### Apoptosis assays

HD-MY-Z KD cell lines (sh*Bmal1*, sh*Per2*, sh*Rev-erbα* and sh*Tnf*) as well as WT cells were seeded in 6-well plates containing RPMI 1640 (Gibco) supplemented with 1% penicillin-streptomycin (Gibco) and 10% foetal bovine serum (Gibco). At around 80% confluency cells were washed once with 1x PBS (Gibco) and counted using a Luna Automated Cell Counter. Cells were seeded in a 96-well plate (Eppendorf) at a concentration of 2500 cells/100 µL medium and incubated for 24 hours at 37 °C with 5% CO_2_. For each cell line three biological replicates and four technical replicates were prepared. After 24 hours incubation, cell media were replaced with fresh medium containing caspase 3/7 (1:2000) and TNF (50 ng/mL) (only for the TNF stimulation experiment). Cell apoptosis was measured using the IncuCyte® S3 Live Cell System Analysis. Cells were scanned every 3 hours with a 10x objective and by using the phase and green image channels. Apoptosis data were normalized with the proliferation data for each cell line.

#### Migration assays

For the migration assay, 35000 cells/well were seeded in a 96-well Essen ImageLock^TM^ microplate (Essen BioScience) and incubated overnight at 37 °C, 5% CO_2_. In the next day, the WoundMaker^TM^ (Essen BioScience) was used to create precise and reproducible wounds. The medium was replaced with fresh medium and the plate was placed in the IncuCyte® S3 Live Cell System Analysis (Essen BioScience). Image acquisition was performed by setting the “scan type” to Scratch Wound and Wide Mode, using the 10x objective. The plate was scanned every hour. Analysis was performed with the scratch wound method in the IncuCyte S3 Software (Essen BioScience).

### Statistical Analysis

The experiments were carried out with at least three biological replicates for each condition. All results are represented as mean ± SEM. Statistical analysis was performed using two-tailed unpaired t-test. A p-value < 0.05 was considered as statistical significant. (*p < 0.05; **p < 0.01; ***p < 0.001).

### Data availability

Data described in this study will be available from the corresponding author upon request. The microarray dataset has been deposited in the ArrayExpress database at EMBL-EBI (www.ebi.ac.uk/arrayexpress) under the accession number E-MTAB-6400.

## Electronic supplementary material

Supplementary Information

Supplementary Table 1
