# Supplementary material for: The reciprocal interplay between TNFα and the circadian clock impacts on cell proliferation and migration in Hodgkin lymphoma cells
Source: Sci Rep. 2018 Jul 31;8:11474. doi: 10.1038/s41598-018-29847-z (PMC6068144; doi:10.1038/s41598-018-29847-z)
Supplement: Supplementary file 1 — Supplementary Information [file 41598_2018_29847_MOESM1_ESM.pdf]

Supplementary Information

The reciprocal interplay between TNFα and the circadian clock impacts on cell proliferation and migration in Hodgkin lymphoma cells

Mónica Abreu[1,2], Alireza Basti[1,2], Nikolai Genov[1,2], Gianluigi Mazzoccoli[3], Angela Relógio[1,2,*]

[1]Charité - Universitätsmedizin Berlin, corporate member of Freie Universität Berlin, Humboldt - Universität zu Berlin, and Berlin Institute of Health, Institute for Theoretical Biology, Germany

[2]Charité - Universitätsmedizin Berlin, corporate member of Freie Universität Berlin, Humboldt - Universität zu Berlin, and Berlin Institute of Health, Medical Department of Hematology, Oncology, and Tumor Immunology, Molecular Cancer Research Center, Germany

[3]Department of Medical Sciences, Division of Internal Medicine and Chronobiology Unit, IRCCS "Casa Sollievo della Sofferenza", San Giovanni Rotondo (FG), Italy

[*]Corresponding author: angela.relogio@charite.de

Supplementary Materials

Table S1 – Pairwise interactions Clock-Immune Genes

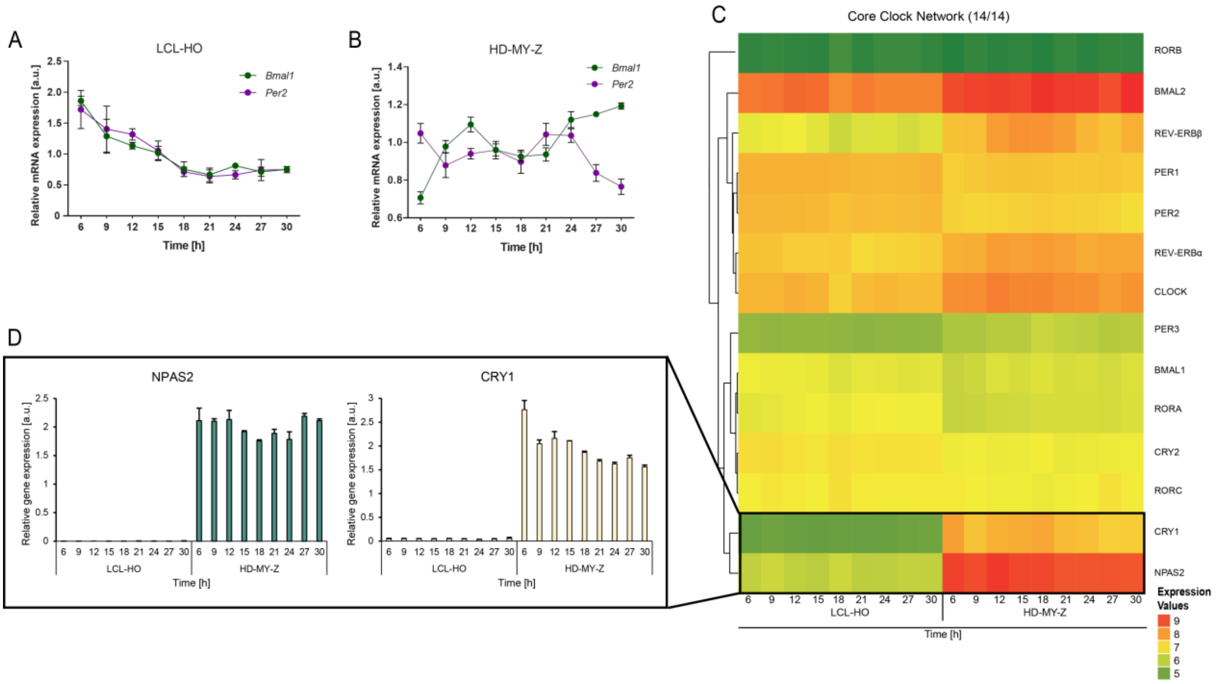

Figure S1 – **LCL-HO and HD-MY-Z have differential circadian phenotypes** Depicted are gene expression values of *Bmal1* and *Per2* for LCL-HO (**A**) and HD-MY-Z (**B**) over 24 h (n=3, mean ± SEM), expression of core-clock genes in the HTA 2.0 for both cell lines (**C**) and (**D**) 24h time-course qPCR for *NPAS2* and *CRY1* genes (n=3, mean ± SEM).

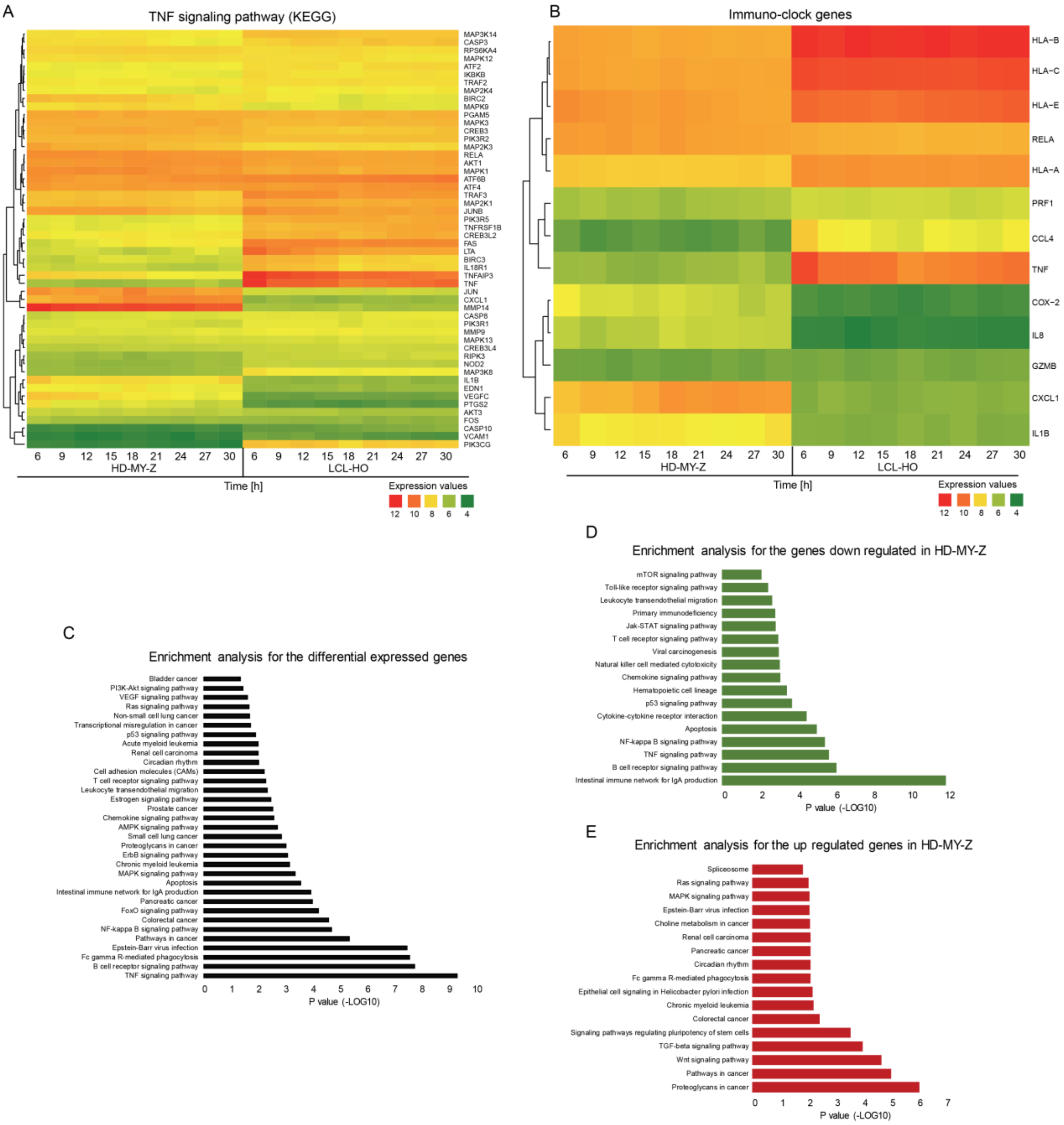

Figure S2 – **The TNF signalling pathway is deferentially expressed in HL**. (**A** and **B**) heatmaps showing the relative expression of the differentially expressed genes in the TNF signalling pathway and immune-clock genes (from Figure 1). (**C**, **D** and **E**) enrichment analysis for KEEG pathways of the differentially expressed genes in LCL-HO and the HL cell line HD-MY-Z.

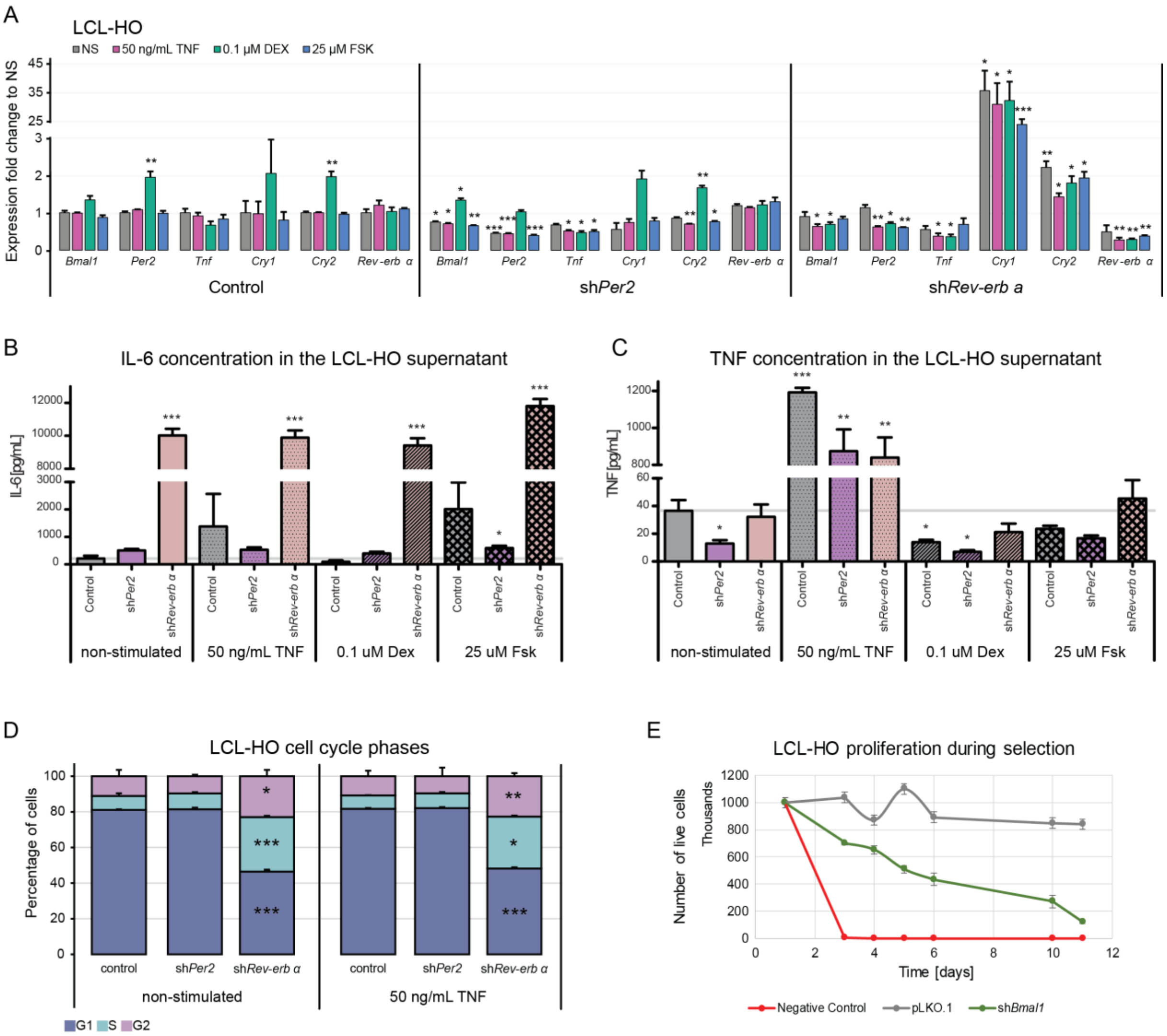

Figure S3 – **TNF affects cell cycle and expression of core-clock genes in LCL-HO cell line.** (**A**) Gene expression qPCR data (n=3, mean ± SEM), (**B**) IL-6 and (**C**) TNF concentration in the supernatant (n=3, mean ± SEM) (**D**) measurements of cell cycle phases (n=3, mean ± SEM) after shRNA KD of *Per2* and *Rev-erb α* and stimulation for 3 days with 50 ng/mL of recombinant human TNF, 0.1 µM of dexamethasone and 25 µM of forskolin. (**E**) proliferation measurements after sh*Bmal*1 (n=3, mean ± SEM). Statistics for the compassion to non-stimulated using unpaired t test: * $p < 0.05$, ** $p < 0.01$, *** $p < 0.001$. LCL-HO WT, LCL-HO transduced with pLKO.1 empty vector and transduced with sh*Bmal1* KD vector proliferation during the selection with puromycin.

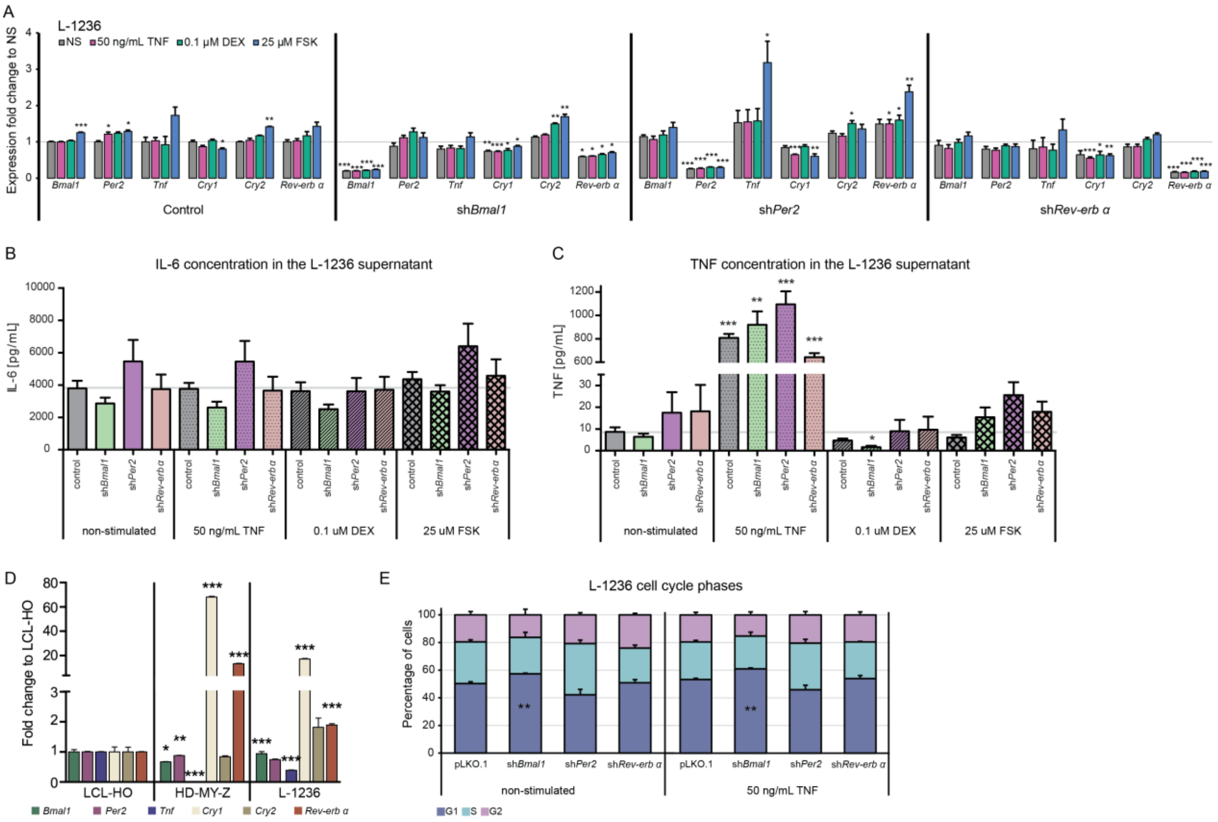

Figure S4 – TNF affects cell cycle and expression of core-clock genes in the HL cell line L-1236. (**A**) Gene expression qPCR data (n=3, mean ± SEM), (**B**) expression levels of core-clock genes and Tnf in HL cell lines, qPCR data (n=3, mean ± SEM) (**C**) IL-6 and (**D**) TNF concentration in the supernatant (n=3, mean ± SEM) (**E**) measurements of cell cycle phases (n=3, mean ± SEM) after shRNA KD of *Bmal1*, *Per2* and *Rev-erb α* and posterior stimulation for 3 days with 50 ng/mL of recombinant human TNF, 0.1 µM of dexamethasone and 25 µM of forskolin. (Statistics for the comparison to non-stimulated using unpaired t-test: * p < 0.05, ** p < 0.01, *** p < 0.001.

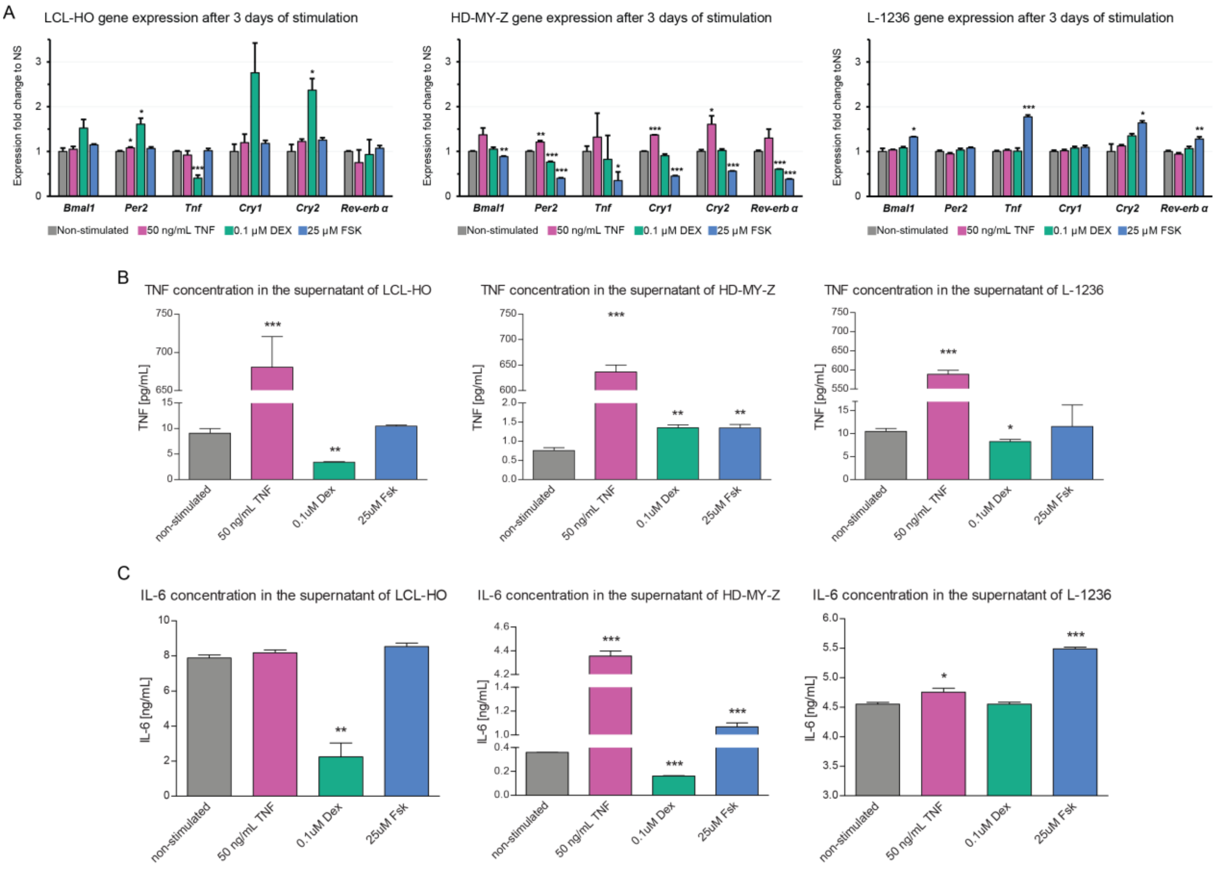

Figure S5 – Overall comparison for the effects of TNF on the core-clock in HL cell lines. (**A**) Gene expression qPCR data (n=3, mean ± SEM), (**B**) TNF and (**C**) IL-6 concentration in the supernatant (n=3, mean ± SEM) for the 3 cell lines (WT) after 3 days stimulation with 50 ng/mL of recombinant human TNF, 0.1 μM of dexamethasone and 25 μM of forskolin. Statistics for the compassion to non-stimulated using unpaired t test: * p < 0.05, ** p < 0.01, *** p < 0.001.

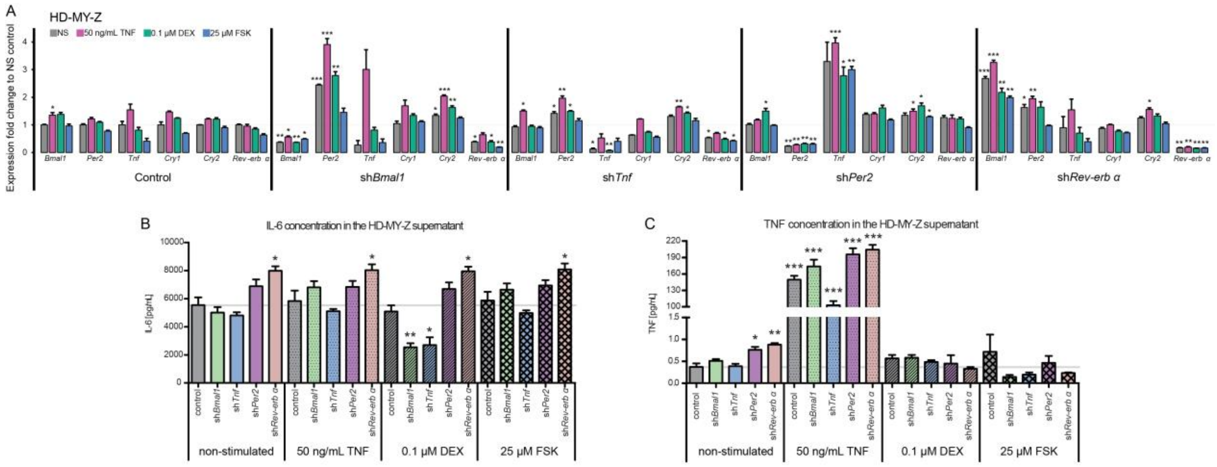

Figure S6 – **The core-clock affects expression of TNF and IL-6 in the HL cell line HD-MY-Z.** (**A**) Gene expression qPCR data (n=3, mean ± SEM), (**B**) IL-6 and (**C**) TNF concentration in the HD-MY-Z supernatant (n=3, mean ± SEM) for the 3 cell lines (WT) after 3 days stimulation with 50 ng/mL of recombinant human TNF, 0.1 µM of dexamethasone and 25 µM of forskolin. Statistics for the compassion to non-stimulated using unpaired t test: * $p < 0.05$, ** $p < 0.01$, *** $p < 0.001$.
